# Supplementary material for: Deep phenotyping unveils hidden traits and genetic relations in subtle mutants
Source: Nat Commun. 2016 Nov 23;7:12990. doi: 10.1038/ncomms12990 (PMC5122966; doi:10.1038/ncomms12990)
Supplement: Supplementary Information — Supplementary Figures 1-10, Supplementary Notes 1-11 and Supplementary References [file ncomms12990-s1.pdf]

Supplementary Figures

Supplementary Figure 1. High-throughput, high-resolution imaging of synaptic sites enabled by microfluidics, computer vision and automated control. a) Top: localization of DA9 motorneuron in *C. elegans*. Bottom shows differences in images of fluorescently labeled presynaptic sites in DA9 imaged from a lateral and a dorsal-down orientation. Scale bar is 10 µm. b) Diagram of the microfluidic chip, blue channel is for cooling liquid flow, green channels are valves and red channels are worm flow and flush channels. Inset shows detail of the imaging channel. c) Diagram showing components of integrated approach. d) Images of animals in head-first (right) and tail-first (left). Top images acquired in the red channel, bottom images acquired in the green channel. e) Classification of dorsal-down (left) vs ventral-down (right) performed by identification of the gut orientation.

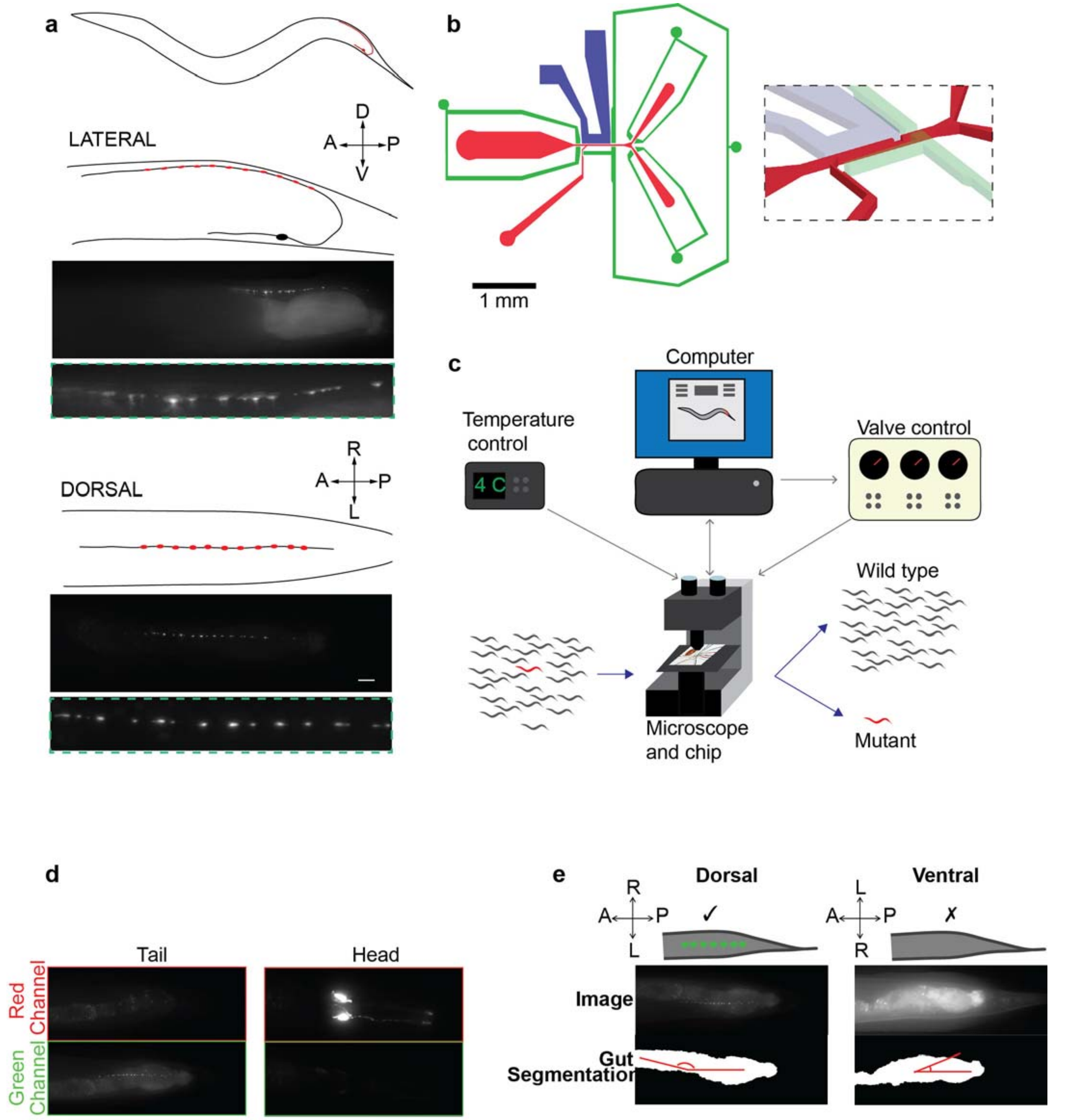

Supplementary Figure 2. Unsupervised synapse detection steps. 1) Maximum projection of acquired z-stack. 2) Mathematical transformations are applied to the original image. 3) and 4) A probability image and a decision image is obtained from the SVM model and the original and transformed images. Bottom shows overlay (red) of detected synapse and original image. 5) Features are computed from the detected synpatic puncta.

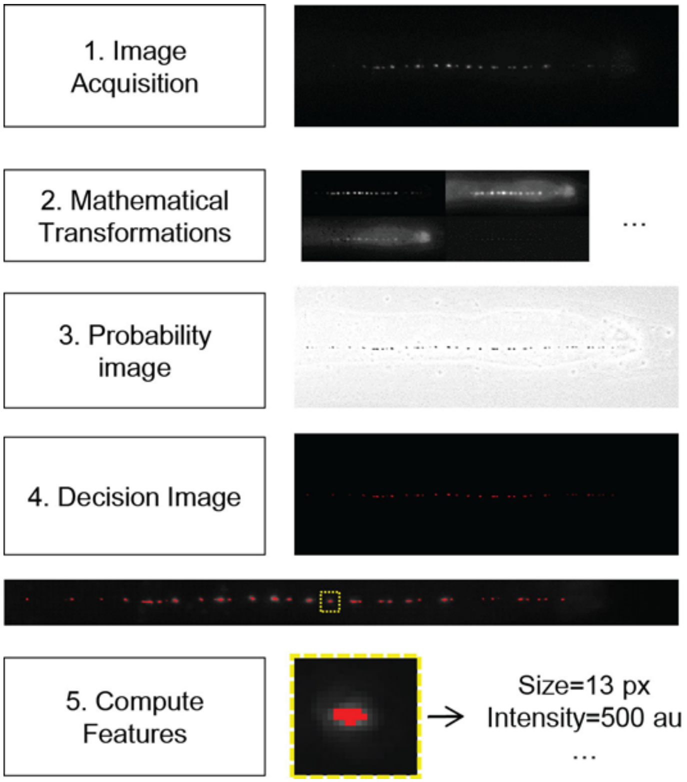

Supplementary Figure 3. SVM, unlike typical segmentation methods, achieves high accuracy in synapse detection. Top shows an original image of synaptic puncta (images on the right are zoom-ins of the image on the left). Second row shows segmentation results with Otsu's threshold. Third and fourth rows show segmentation results with a threshold 2.5 and 5 times larger than Otsu's threshold, respectively. Fifth row shows results with k-means segmentation. Last row shows results with the method employed here, Support Vector Machines (SVM).

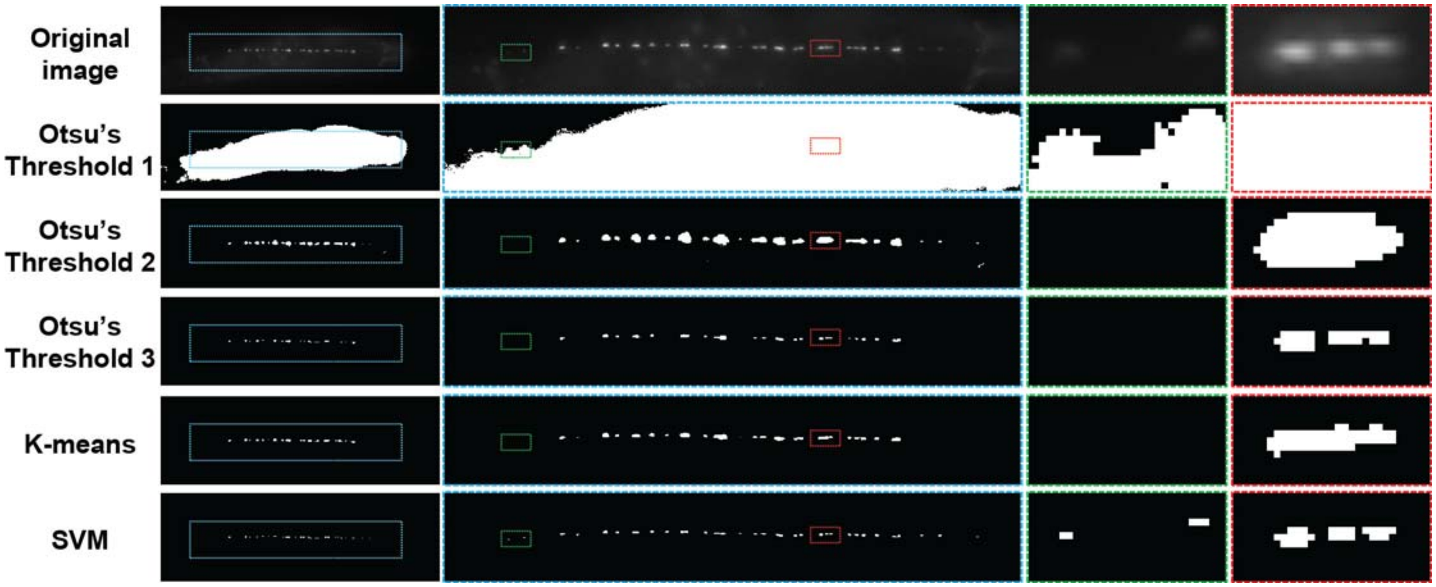

Supplementary Figure 4. Screening and clonal population characterization procedure. During the screening step, single animals are phenoyped and isolated. Clonal populations generated from isolated mutants are then studied and compared to wildtype populations to determine phenotype severity and significantly different characteristics. Mutants can then be analyzed *via* downstream methods, i.e., whole-genome sequencing, SNP mapping, phenocopy and rescue experiments, complementation tests, etc.

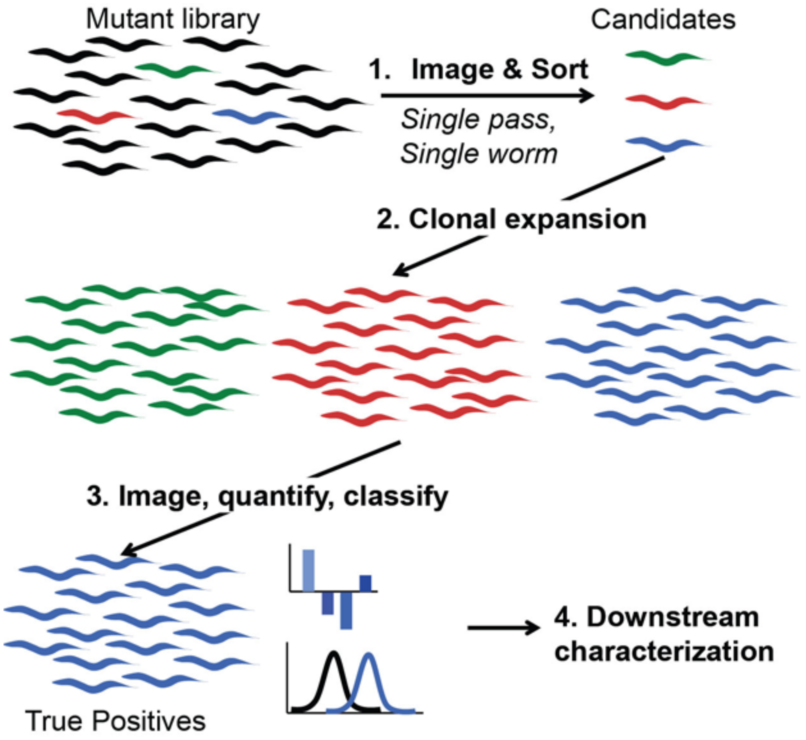

Supplementary Figure 5a. Fluorescent images of all genotypes used in this work. 4 images per genotype. Known mutant collection. Wildtype scale bar is 20 μm.

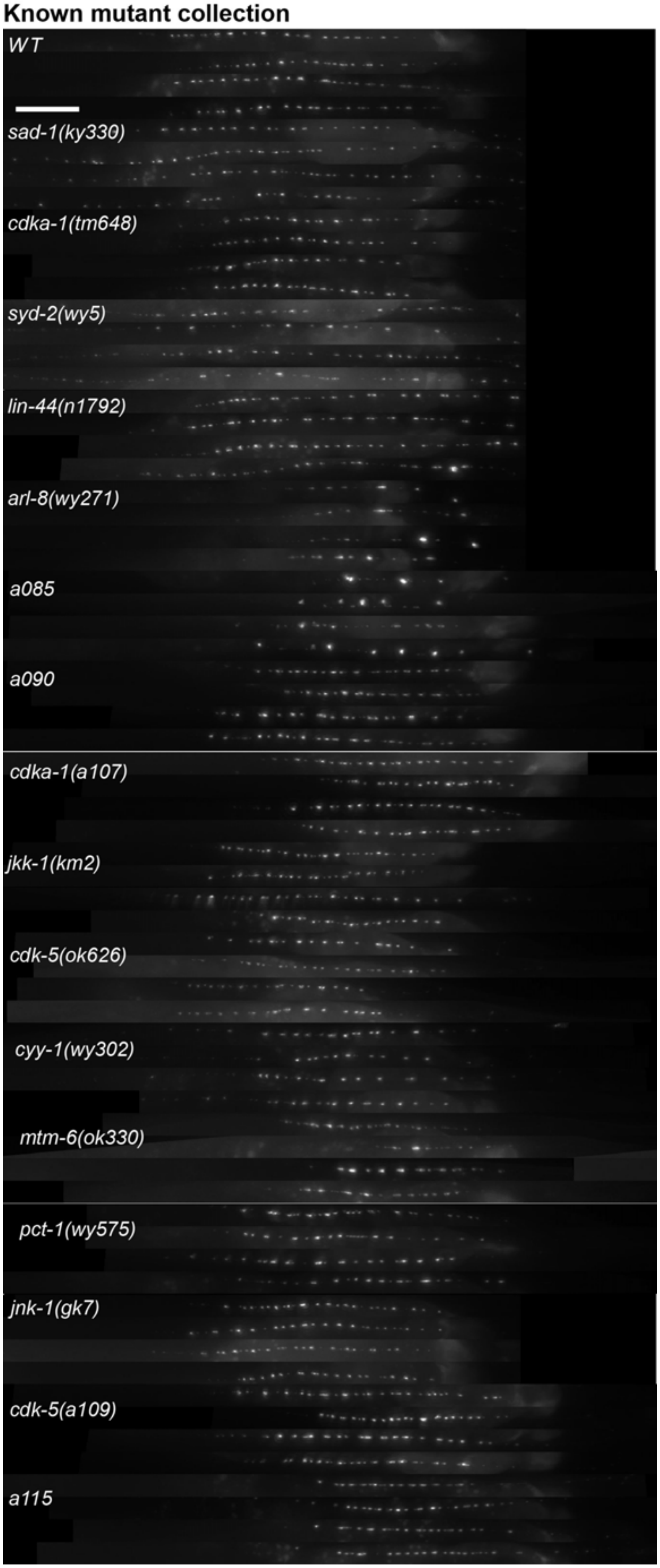

Supplementary Figure 5b. Fluorescent images of all genotypes used in this work. 4 images per genotype, Isolated mutant collection

Isolated mutant collection

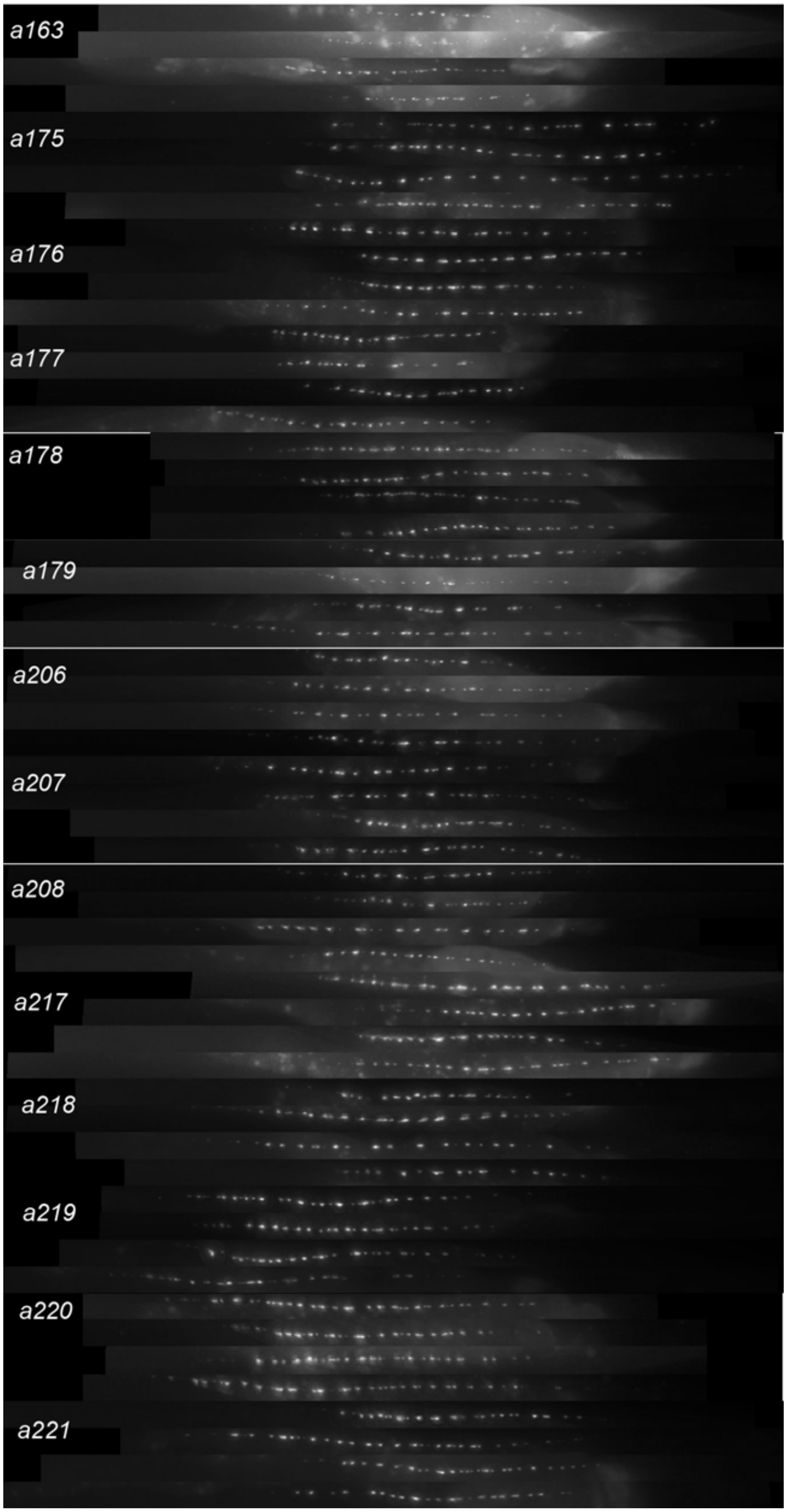

Supplementary Figure 5c. Fluorescent images of all genotypes used in this work. 4 images per genotype. Top: Isolated mutant collection, continued. Bottom: *sax-2* alleles

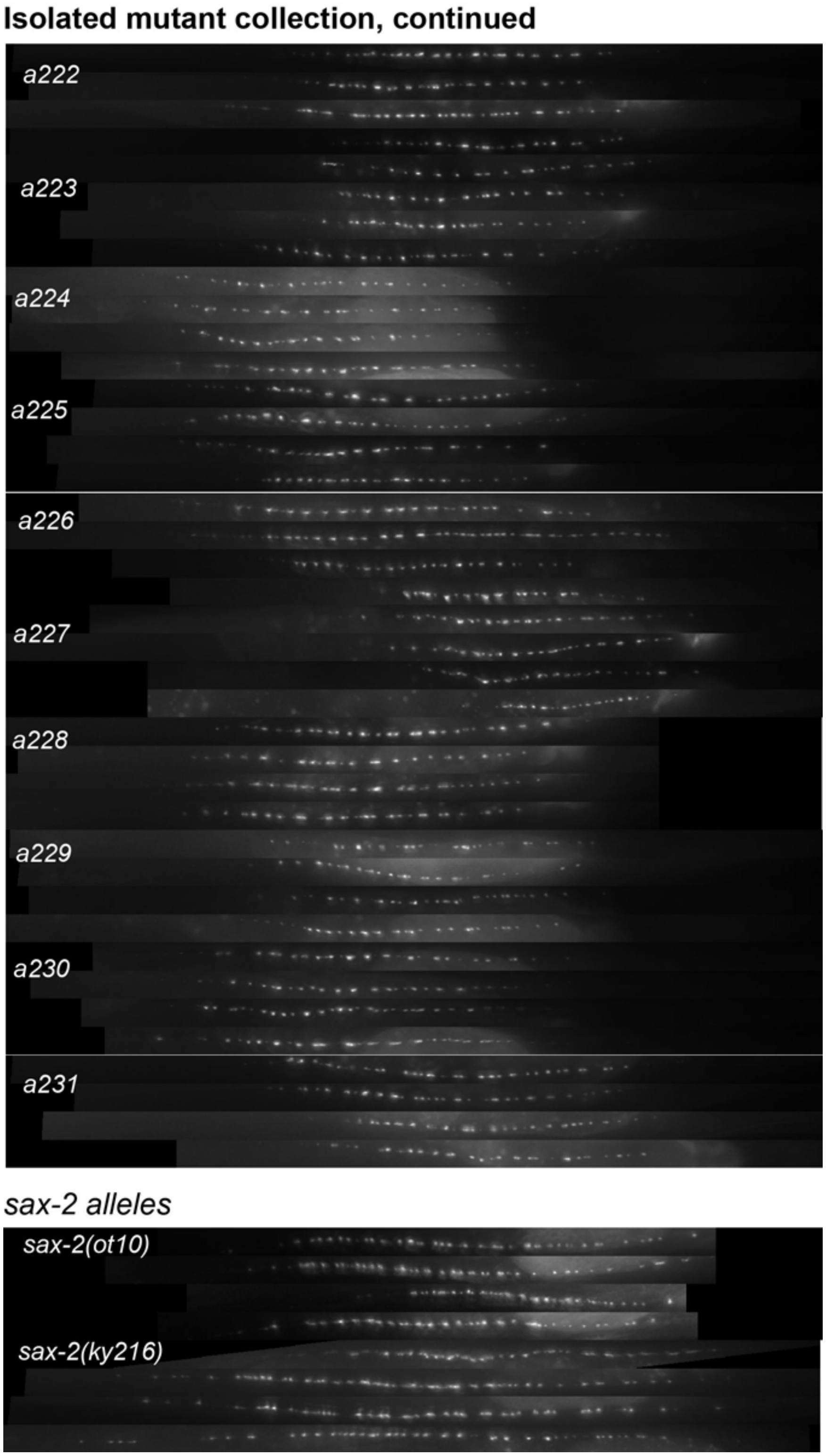

Supplementary Figure 6. Sources of mutant phenotypic differences are revealed by feature selection from logistic regression models. Relevant features obtained from step-wise logistic regression (from most relevant to least relevant left to right). Line plotted on right axes represents the model deviance reduction by adding each plotted feature.

See next page.

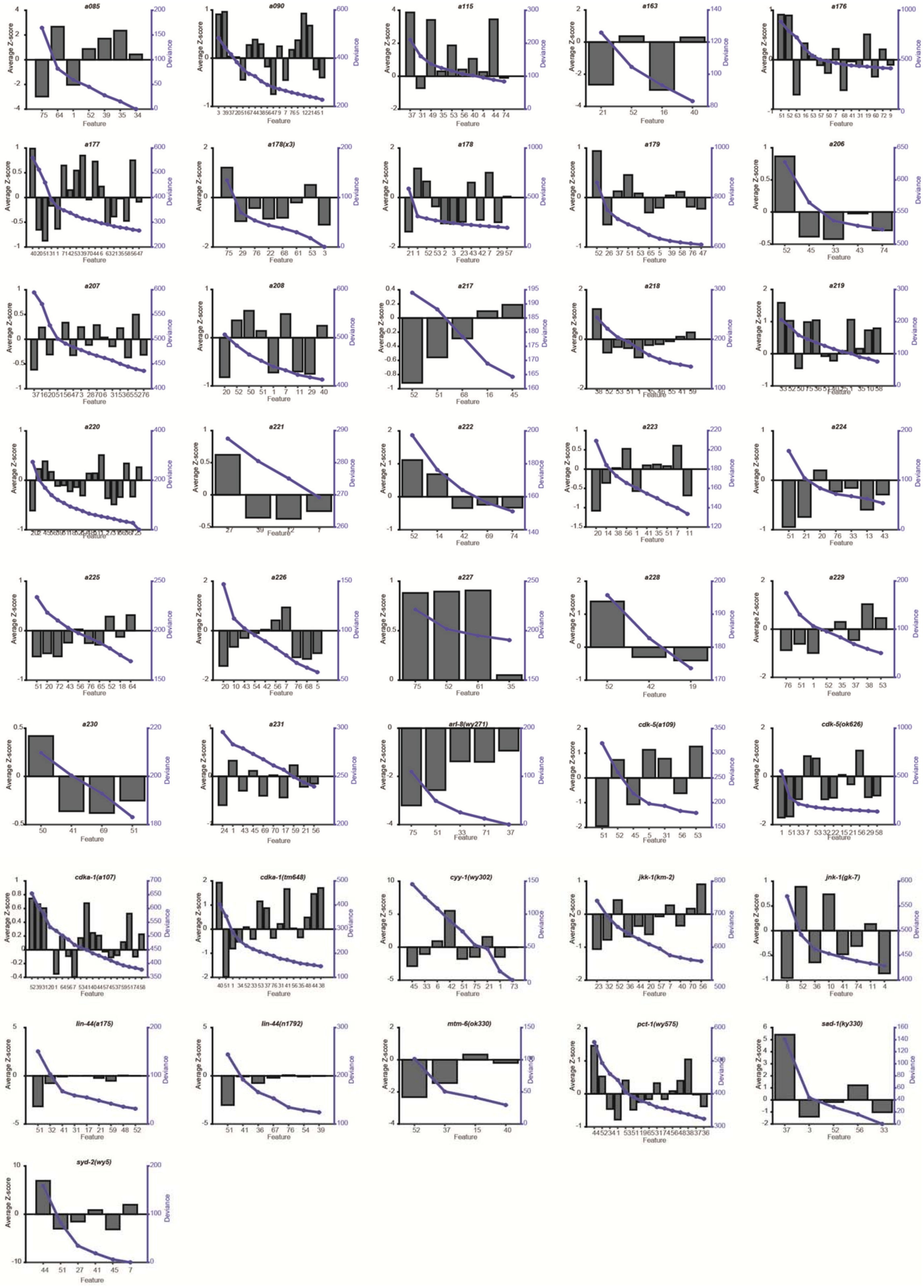
a085
a090
a115
a163
a176
a177
a178(x3)
a178
a179
a206
a207
a208
a217
a218
a219
a220
a221
a222
a223
a224
a225
a226
a227
a228
a229
a230
a231
arl-8(wy271)
cdk-5(a109)
cdk-5(ok626)
cdka-1(a107)
cdka-1(tm648)
cyy-1(wy302)
jkk-1(km-2)
jnk-1(gk-7)
lin-44(a175)
lin-44(n1792)
mtm-6(ok330)
pct-1(wy575)
sad-1(ky330)
syd-2(wy5)
Average Z-score
Deviance
Feature

Supplementary Figure 7. Model performance is independent of number of features. AUC for ROC curves is not correlated to the number of features in each model.

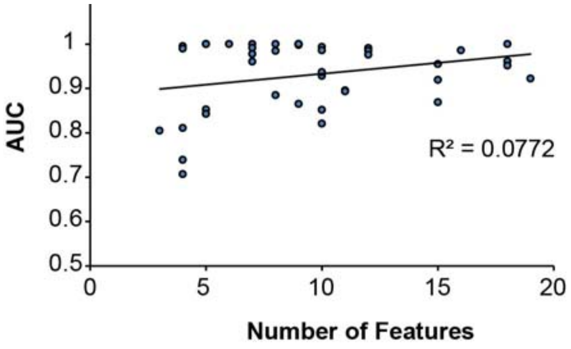

Supplementary Figure 8. Top: Swimming behavior of *sax-2* alleles. Mutant *a178* displays a robust defective swimming behavior. The fraction of animals swimming is drastically reduced as compared to wildtype. Alleles *ot10* and *ky216* display a less drastic reduction in swimming locomotion. **Bottom:** complementation tests suggest *a178* is in fact an allele of *sax-2.*

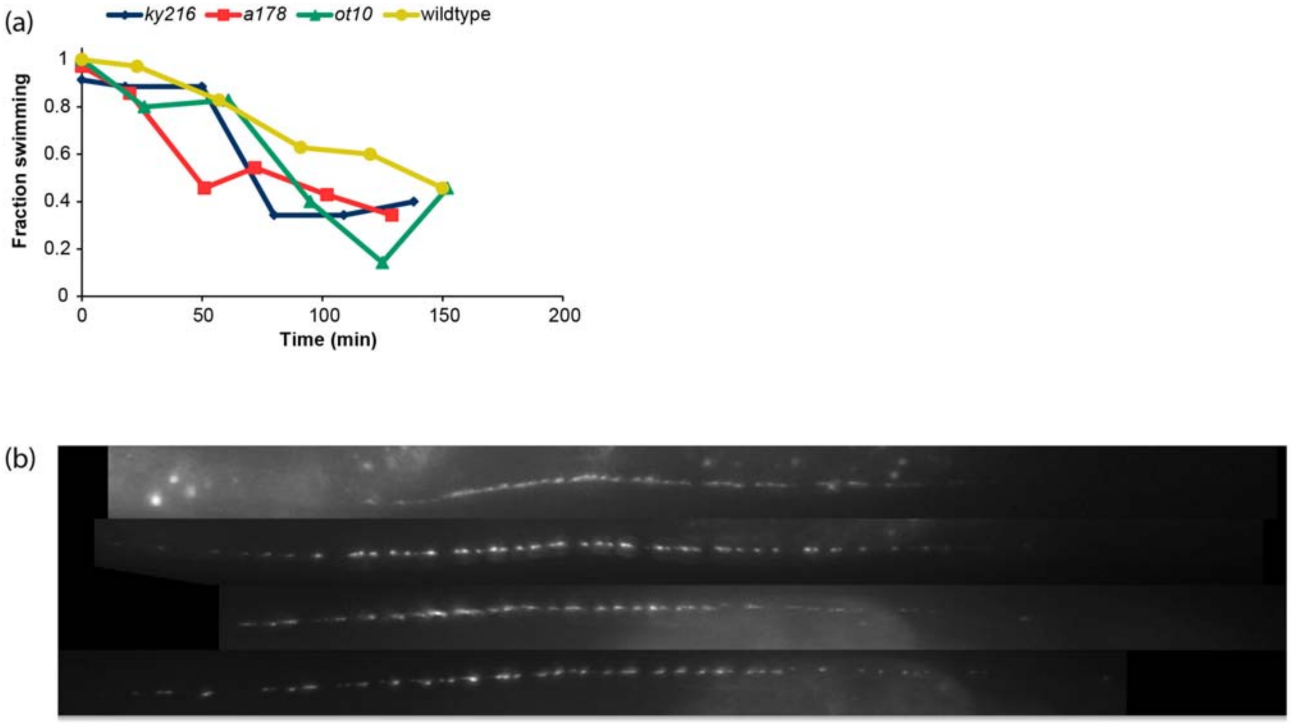

Probability of having *a178* phenotype

Mean	**0.61**
Std. Dev.	**0.40**
Image 1	0.07
Image 2	0.99
Image 3	0.81
Image 4	0.56

Supplementary Figure 9. 5-fold cross-validation accuracy for BF(red) and SWLR(blue) models. Shaded regions represent the standard deviation from the 5-fold cross-validated model accuracy**.** SWLR models result in higher accuracy without sacrificing model performance.

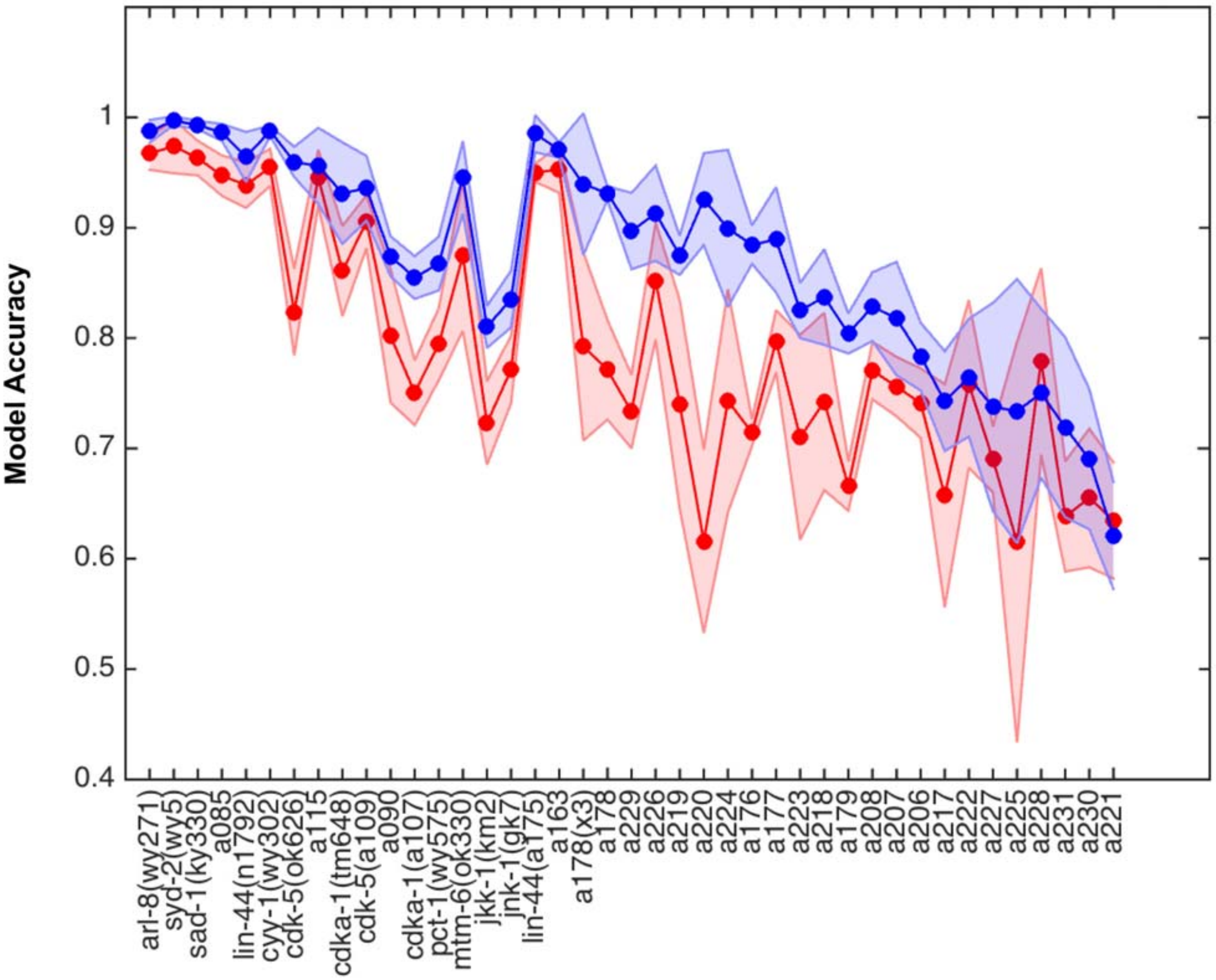

Supplementary Figure 10. Visualization tools aid in identifying differences in average morphometric features. Sources of morphological differences against wildtype for known and found mutants. Pie charts represent the weighted difference against wildtype for the features selected by the logistic regression model. Features were classified into seven categories. Similarities between related genotypes are evident (eg. alleles of *lin-44)*

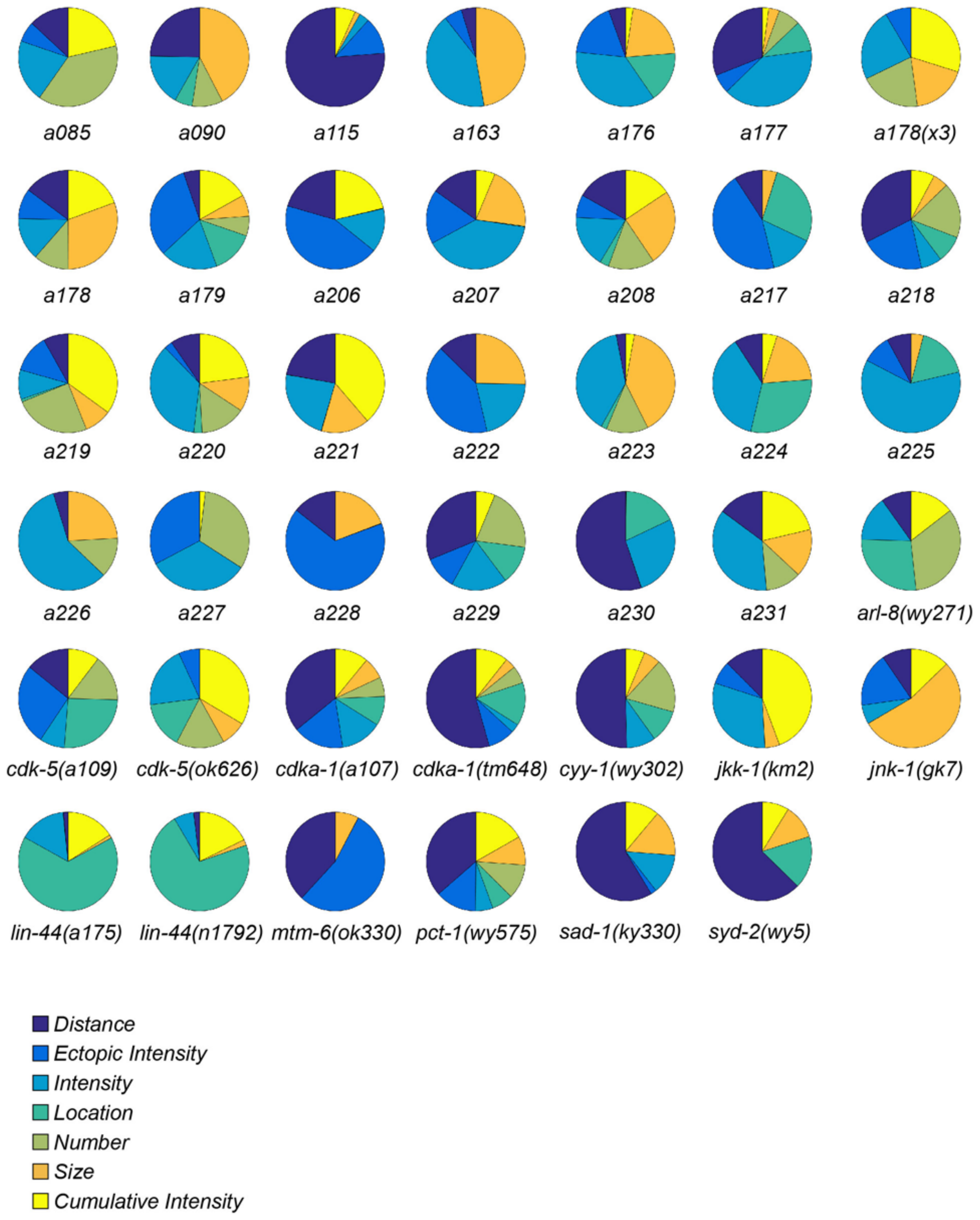

Supplementary Notes

1. High-throughput imaging and phenotyping

Our automated screening platform incorporates microfluidics (for worm handling, imaging, and sorting), external support hardware, automated control of image acquisition and external components, and computer vision tools for unsupervised annotation of images. Here in order to achieve the data extraction capabilities required to identify subtle alleles, we further adapted this system for higher sensitivity and specificity of synapse detection (**Supplementary Figs. 1, 2, 3**).

1.1. Device design and operation

A microfluidic device provides a platform for easy worm handling and imaging without the need for anesthetics [1]. The microfluidic device (**Supplementary Fig. 1b)** contains on chip valves that enable image acquisition, analysis and sorting of a single worm at a time. The flow channels incorporate an inlet for worm injection, a narrow channel for individual worm imaging, two outlets for worm exit and a flush channel that is used to inject liquid and push worms towards one of the exits after analysis. The imaging channel can be isolated from the exits and the inlet by on-chip valves. These valves are closed-end channels filled with a water/glycerol solution, which can be pressurized, deforming the membrane between them and the flow channels. Once a worm is trapped in the imaging channel, image acquisition begins, which requires worm immobilization. A cooling channel adjacent to the worm imaging channel is used to flow a cooled liquid, which reduces the temperature in the main imaging channel, and thus immobilizes the worm for the amount of time required for imaging [2], typically 2 seconds.

One novelty of our microfluidic device (**Supplementary Fig. 1b)** is that it positions animals in a dorso-ventral orientation, greatly improving the image quality of synaptic sites. Since synaptic patterns are located on the dorsal cord, images acquired in a lateral orientation (the conventional configuration for analysis [3]) contain significant image aberrations that prevent extraction of detailed phenotypic information (**Supplementary Fig. 1a)**. The microfluidic device design was optimized to improve the bias towards a dorsal-down, tail-first orientation by incorporating a straight imaging channel with a small vertical step at the stopping position on the imaging channel [4] and no inlet pillars [5] (**Supplementary Fig. 1**). Only worms that come in to the channel in the correct orientation are imaged. Worms that come in head-first are identified due to the presence of a red fluorescent gene reporter in the head neurons. A dual CCD camera is used for simultaneous two-color imaging. Additionally, dorsal vs. ventral-down orientation is detected based on the shape of the gut.

It has been shown that worm orientation can be biased to a lateral orientation by adding curves or bends to microfluidic channels [4]. With the aim of improving the bias towards a dorsal/ventral orientation, we use a straight channel with a small step at the end (**Supplementary Fig. 1c**). The inclusion of the step in the straight channel was observed to practically eliminate the occurrence of laterally oriented animals. An added advantage provided by the inclusion of the step at the end of the imaging channels is that by reducing the available cross-sectional area, the tail of the worm is pushed closer to the cover-slip thus providing better imaging conditions and improving image quality. The inclusion of pillars in the loading area has been suggested to increase the loading of worms in microfluidic chips towards a head-first orientation [5]. The loading area of this chip is therefore designed with no pillars or bends.

The imaging cycle starts with the entry of a worm from the inlet chamber into the imaging channel. Once a worm is detected in the imaging channel, the entry and positioning valves are closed and image acquisition starts after the time required for immobilization by cooling has passed. During the imaging step, a stack composed of twenty images, one micron apart in the z direction, is acquired. Obtaining images in different focal planes is important since synapses are found in several focal planes. The images are then analyzed and a decision is made on whether the worm trapped in the imaging channel belongs to the wild type population or if it is a putative mutant. The animal is then sorted to the wild type or mutant exit for disposal or collection, respectively. Once a worm is sorted, another worm enters the imaging channel and the cycle starts again. In this way, image acquisition, analysis and decision making is performed in line while a worm is in the imaging channel. Integrating microfluidics with off-chip hardware, image processing and automation allows continuously running fully automated high-throughput genetic screens without any supervision.

The device operation is performed with the help of a pneumatic box that provides control over the pressure exerted on each of the lines connected to the microfluidic chip, as shown in **Supplementary Fig. 1**. Flow to the worm inlet and flush channels are pressure-driven under a controlled set point, typically 2-4 psi for the worm

inlet and 4-5 psi for the flush channel. This pressure controller is additionally used to actuate each on-chip valve individually [2]. Off-chip pinch valves allow additional on/off control of the pressure-driven flows by actuating directly on the inlet tubing. Cooling liquid flow is driven by a peristaltic pump that feeds an intermediate volume capacitor to ensure it remains undisturbed. The temperature of the cooling liquid, a mixture of glycerol and water, is adjusted by a Peltier cooler to ~ 4° C. The pressure box and off-chip valves are actuated with a Graphical User Interface (GUI) in Matlab (® Mathworks) that controls the synchronization of valve actuation and flows in each stage of a worm imaging and sorting cycle. Other off-chip components including the microscope stage and camera are also controlled with the GUI. With this setup, approximately 130 worms are comprehensively phenotyped per hour.

1.2. Detection of worm orientation

Image processing and machine vision algorithms enable the identification of the orientation of animals in the imaging channel, and those in an inadequate orientation for imaging are automatically discarded through the wildtype exit. An important first step in the automated screening or imaging algorithms is the identification of the worm orientation. Animals can enter the imaging channel in a head-first or a tail-first orientation. Additionally, animals can be positioned in a ventral-down or dorsal-down orientation. The design of the device practically eliminates the occurrence of laterally oriented animals. Since the presynaptic domain of DA9 motorneuron is located in dorsal side of the tail, the system is trained to identify and analyze only animals in the right orientation (i.e., tail-first and dorsal-down). All animals that enter the imaging channel in the wrong orientation are not analyzed and directed towards the wild-type exit.

Worm orientation identification is a two-step process. In the first step, a classifier determines whether the portion of the worm in the field of view is a head or a tail. If the decision is a head, the worm is discarded. If the decision is a tail, the system proceeds to the second step where the animal is classified as either being in a ventral-down or a dorsal-down orientation. Animals classified as ventral-down are discarded. These classification steps are performed on an image acquired as soon as the worm has entered the imaging channel. In order to expedite the process, the image is acquired before the time necessary for immobilization by cooling has passed. In this way, no time is consumed in worms that will not be analyzed.

a) Head/Tail Classification

As previously mentioned, images are acquired with a dual-color camera with a green and a red channel. Animals contain a red marker in the head, while synaptic puncta are labeled green, as shown in **Figure S1d**. A simple SVM was built to detect head vs. tail by taking into account the intensity of the green and red channels. The SVM was implemented with the open-source library libsvm [6].

b) Dorsal/Ventral Classification

Once an animal in a tail-first orientation is detected, the system proceeds to a second step classifier, where the tail image is classified as either dorsal-down or ventral-down orientation. Since images are acquired with an inverted microscope, and the presynaptic sites of interest are located along the dorsal cord, the desired orientation is dorsal-down. Differentiation between dorsal-down and ventral-down orientation was accomplished by identifying the shape of the intestine. As shown in **Supplementary Fig. 1e** the most anterior section of the gut in the field of view turns upward when the animal is imaged from a dorsal-down orientation, and downward when the animal is imaged from a ventral-down orientation. This anatomical feature facilitated the distinction of the worm orientation without requiring additional fluorescent markers. It is important to mention that relying in the presence of GFP signal from the presynaptic sites is not ideal since some mutants might show a decreased or increased GFP signal in both the dorsal and ventral side (for example, if they exhibit mislocalization of synaptic puncta to the dendritic domain).

In order to determine whether an animal tail was in the correct orientation, a three-step process was implemented, explained next:

1) Gut segmentation from an initial single snapshot: The gut is segmented by implementing Otsu’s method thresholding.
2) Computation of the angle at which the gut is positioned (with respect to a horizontal line): the angle is quantified as the angle between the major axis of the ellipse containing the segmented gut and the horizontal line (regionprops in MATLAB).
3) Decision of ventral-down, dorsal-down or further processing required:
    a. If the angle is below 1, the animal is classified as ventral-down and discarded

b. If the angle is above 2.5, image acquisition starts after the necessary time for cooling
c. If the angle is above 1 and below 2.5, the animal is cooled down and a second image is acquired for classification of dorsal-down or ventral-down. The image is segmented and the angle of the gut is quantified. If the angle of the new image is above 1, the animal is analyzed, otherwise it is discarded.

1.3. Synaptic Pattern Phenotyping

To extract morphological data from synaptic patterns, we first detected the fluorescently labeled puncta, from images, which typically contain noise and gut autofluorescence. Puncta detection from dorsal views of the DA9 axon was performed by image classification using a support vector machine (**Supplementary Figs. 4, S5**). Importantly, images include exclusively the DA9 dorsal axon. As previously mentioned, many of the distinct mutants display presynaptic sites in the dendrite (located on the ventral side). Analyzing animals from a dorsal orientation (rather than a lateral orientation) provides images with better quality, thus enabling extracting more reliable information for synaptic patterns (**Supplementary Fig. 1**). However, the identification of dendritic phenotypes is inaccessible in this approach. Nonetheless dendritic mislocalization mutants do not classify as subtle phenotypes, whose identification is the goal in this work. Once synaptic sites from the dorsal view are detected, descriptors to build an in-depth phenotypic profile are quantified. These include average synapse size, intensity, number of puncta, and length of the synaptic domain. We also include other less intuitive descriptors that can provide additional information to describe synaptic patterns (**Supplementary Fig. 1c**). Examples of these descriptors ("meta-features") include homogeneity of synapse size, heterogeneity amongst reporter distribution between synapses, or inter-punctal spacing variance. In total we compute 76 different metrics that makeup an integrative multidimensional phenotypic profile.

Synaptic Puncta Detection

In the SVM training step, each image has a corresponding label or ground-truth image. In the ground truth image, each pixel is manually labeled as synapse or non-synapse, thus providing positive and negative samples. In addition, the training step requires the same pre-established set of features for each image in the training set. The model used by the SVM is constructed by optimizing the separation between these two classes. The classifier can then use this pre-built model to make decisions on any new image to detect the presynaptic sites.

The classifier used is a one-parameter linear SVM. The cost parameter was found by brute-force grid search with a 5-fold cross-validation, and giving a larger error weight (2x) to false negatives than false positives. Obtaining the precise synaptic puncta size is one of the main goals of the phenotypic profiling performed in this work and thus the puncta detection accuracy is extremely important. Ensuring that all the pixels in a synapse are classified as such is more likely to occur when the error weight given to false negatives is larger, although this increases the risk of false positives. These, however, are easy to identify since they typically do not show up on the dorsal cord or in the synaptic domain. Inspection of the results of synapse detection with images not utilized for the training set showed that the false positive rate is negligible. The performance of the linear SVM model on the training set is:

- False Positive Rate: 0.85%
- True Positive Rate: 92.54%
- False Negative Rate: 7.46%
- True Negative Rate: 99.14%

The training set includes a total of 255,000 negative points and 13,151 positive points (i.e. pixels), coming from 85 different images of 6 different genotypes (WT, *jnk-1, jkk-1, sad-1, syd-2, mtm-6*).

Nonetheless, an outlier detection algorithm was developed to remove potential false positives present in regions outside of the archetypal presynaptic pattern (see supplementary information).

The original image used for synapse detection is the maximum projection of the z-stack acquired from the green channel, while the features are transformations of this original image as well as the maximum projection of the z-stack obtained from the red channel. Presynaptic sites are fluorescently labeled with GFP and thus appear in the green channel while fat globules and gut auto-fluorescence appear, to a different degree, in both channels. Synapse detection was performed with a linear SVM (LIBLINEAR, [7]), where each pixel is classified as either synapse or non-synapse. The features included in the model for training and testing are included next.

1) Original image
2) Gradient image

3) Original image convoluted with a 2x110 matrix
4) Difference of Gaussian low pass filters with size and standard deviation of [25,0.1] and [25,1.5]
5) Difference of Gaussian low pass filters with size and standard deviation of [25,0.1] and [25,5]
6) Original image processed with a circular averaging filter of radius 5
7) Local standard deviation of original image with a neighborhood size of 5
8) Original image processed with a circular averaging filter of radius 11
9) Local standard deviation of original image with a neighborhood size of 11
10) Laplacian of Gaussian filter of size 15 with standard deviation 0.1
11) Original image eroded with a diamond structural element of size 4
12) Ratio of green image over red image, called "ratio image"
13) Gradient of ratio image
14) Ratio image convoluted with a 2x110 matrix
15) Difference of Gaussian low pass filters with size and standard deviation of [25,0.1] and [25,1.5] of ratio image
16) Difference of Gaussian low pass filters with size and standard deviation of [25,0.1] and [25,5] of ratio image
17) Ratio image processed with a circular averaging filter of radius 5
18) Local standard deviation of ratio image with a neighborhood size of 5
19) Ratio image processed with a circular averaging filter of radius 11
20) Local standard deviation of ratio image with a neighborhood size of 11
21) Laplacian of Gaussian filter of size 15 with standard deviation 0.1 applied to ratio image
22) Ratio image eroded with a diamond structural element of size 4

Outlier Detection

After the synapse detection step, an outlier detection algorithm was implemented to detect pixels classified as synaptic puncta that lie outside the normal synaptic domain. The occurrence of such false positives, however, was extremely rare. The outlier detection algorithm was based on the distance of each puncta centroid to the predicted location by a line fit to all the puncta. The process is a two-step procedure:

1) The first line is fit to the 2/3 of the puncta closest to the mean y location. After a line is fit, the displacement from the predicted y location is computed. Those points with an error above 40 pixels are erased.
2) A second line is fit to the first half of the remaining puncta (the most anterior puncta are usually positioned in a straight line). Those points with an error in the y axis above 20 pixels are erased.

2. Screening and Putative Mutant Ranking

To identify true mutants while maintaining the lowest possible false positive rate, we built a stringent classifier to detect animals far from the established wildtype phenotypic mean. A randomly mutagenized population typically displays a significantly larger degree of variability than a wildtype population, since mutations are introduced that can affect the growth rate, the animal size, their feeding patterns, etc, which all affecting the phenotypic output to a certain degree. To avoid screening these effects inadvertently by an outlier method, we first aimed at identifying mutants that displayed subtle changes in a few targeted characteristics, rather than taking an integrative multiparametric approach.

To reduce the occurrence of false positives and prevent overfitting, we restricted the identification of mutants to six descriptors we considered most relevant in detecting subtle differences in synapse size and density: average synapse size, average synapse size ignoring single pixel puncta, length of synaptic domain, homogeneity of synapse sizes, size of the largest puncta, and percentage of synapses smaller than six pixels. For these descriptors a low and a high threshold were selected based on the values observed for the wildtype population, allowing a maximum false positive rate of 1.5%. We then performed an unbiased forward genetic screen by imaging, phenotyping, and sorting a mutagenized population. The screens were conducted in a several steps. In the first step, we identify putative mutants with higher likelihood of carrying a phenotype (of interest). We then analyzed populations from the putative mutants with a higher likelihood of displaying phenotypic differences in synaptic patterning. Although the first step focuses on a few characteristics (quantified without user input), the second characterization step is an integrative phenotyping where the most relevant differences are detected through the SWLR model construction.

In the first screening step, animals were isolated if any of the computed descriptors fell outside of the established range.

Three rounds of screening were performed.

In total ~4100 worms were screened and 155 worms were sorted as mutant (~3.7%). The same rules for sorting mutants were used in all screens (numbers are pixels, except where noted, 100 pixels = 16.125 um):

- average synapse size: low threshold = 4, high threshold = 13
- average synapse size ignoring single pixel puncta: low threshold = 4.5, high threshold = 14
- length of synaptic domain: high threshold: 800
- homogeneity of synapse sizes: high threshold= 80 (variance / size^2, ignoring single pixels, see feature #4)
- size of the largest puncta: high threshold: 40
- percentage of synapses smaller than six pixels: high threshold = 75 %

- First screen:

Five putative mutants were isolated in the first round of pilot screens. These were re-scored by imaging an age-synchronized cloned population and computing all the phenotypic descriptors for comparison with wildtype. Animals with large average differences in any of the metrics were retained. Four were discarded as false positives and one was retained as a putative mutant for further analysis, *a163*.

- Second screen:

80 putative mutants were found in the next round of screening sessions, 62 were viable animals producing viable offspring. Given the high number of the remaining putative mutants, a first round of re-scoring was performed on the progeny of the isolated mutants by imaging 10-15 worms in the microfluidic device, and computing the phenotypic descriptors. By applying the classifier used during the screen, with the addition of two features (number of puncta and location), each putative mutant was ranked according to the fraction of animals classified as mutants on the first round of re-scoring. From these, the 8 highest ranked putative mutants were retained for further analysis (*a175,a176,a177,a178,a179,a206,a207,a208)*

- Third screen:

70 putative mutants were found in the next round of screening sessions, 57 were viable. Given the high number of the remaining putative mutants, a first round of re-scoring was performed on the progeny of the isolated mutants by imaging 10-15 worms in the microfluidic device, and computing the phenotypic descriptors. In this last round, each feature was tested for statistical significance difference (anova multiple comparison test) against all remaining 57 populations (56 mutants and wildtype). Each population was scored by the feature that most differed from all remaining populations (i.e., the number of population it was significantly different from). For example, feature 22 for mutant *a220* is significantly different from 56 populations, this feature is the one with the largest number of significant differences, thus its score is 56. All mutant populations were scored in this manner, and the top 15 (15 populations with the largest scores) were retained. These include: *a217, a218, a219, a220, a221, a222, a223, a224, a225, a226, a227, a228, a229, a230, a231.*

3. Data Normalization

Since whole-population imaging experiments were performed at different times, and due to experimental variation, as well as potential changes in the experimental setup, light source, etc, all data was normalized to a wildtype population imaged in the same round (i.e., within two weeks of each other, under the exact same conditions). For each imaging round, a wildtype population was also imaged. All data sets were then z-scored as follows: Each data point for each mutant population was normalized as:

$$z_{i,j}^{k} = \frac{x_{i,j}^{k} - \mu_{i,wildtype}^{k}}{\sigma_{i,wildtype}^{k}}$$

where i = feature (1 – 76)

j = mutant population

k= imaging round (where both j and wildtype were acquired).

4. Step-wise logistic regression

Data was normalized as explained. Starting with a model with no variables, these are added and removed in a stepwise manner. Variable significance was assessed via the likelihood ratio chi-square test. The improvement of the model is analyzed at each step, as determined by the p-value of G, where G represents the difference in deviance between the original model and the model with the new variable included (or removed). The p-value is computed from a χ^2 tail test. When significant improvement to the model is achieved after including an extra variable ($p<0.05$) or after removing one variable ($p<0.1$), variables are added or removed respectively. In the first step, the addition of a single variable is compared to the null model (i.e., with only a constant). [8, 9]

5. *a178* outcrossing and genotyping

Mutant *a178* was crossed with wildtype N2 strain. 4 individuals from the next generation (F1) were picked to individual plates and let to self-reproduce. From the next generation (F2), 36 individuals (with the marker) were picked to individual plates, and let to self-reproduce. From these plates, 12 that were homozygous for the marker were selected, age-synchronized and phenotyped in the same manner as all the mutant populations. These were then scored for a178 phenotype. The process was repeated 3 times. As shown in the main text and **Figure 4**, *a178(x3)* is the only population (out of all 41) that phenocopies *a178* according to the logistic regression models.

Genomic DNA isolation and sequencing library preparation:

Genomic DNA was isolated from *C. elegans* grown on two 9cm NGM plates using Qiagen Gentra Puregene Kit (cat. nos. 158667) following the supplementary protocol for nematodes and purified using Zymo Quick-DNA universal kit (cat. nos. D4068). The DNA libraries were prepared by using Illumina Nextera DNA kit (cat. nos. FC-121-1030) and indexes (cat. nos. FC-121-1011) following the standard protocol. The prepared libraries were sequenced at 100 bp paired-read by Illumina Hi-seq 2500 using the rapid-run mode. Reads were aligned to the N2 reference genome (https://www.wormbase.org/ WS248) using BWA[10], SAMtools[11] and Picard[12] software. The variants were called by GATK[13, 14] and annotated using SnpEff[15].

SNP mapping:

Inbred lines for mapping were generated by crossing twice-outcrossed *a178* (N2 background) with CB4856 (Hawaiian strain). SNP Mapping was performed in accordance with Davis et al. [16]

***sax-2* phenocopies:** *sax-2(ky216)* and *sax-2(ot10)* were used for phenocopy studies. These alleles were crossed with the marker strain and genotyped by PCR and Sanger sequencing. The model developed for *a178* was then used to test phenotypic similarity to wt and *a178*. Swimming assays for these alleles also confirmed behavioral phenotypes exhibited by *a178.*

6. Artificial images construction

The features included in the artificial images are exclusively those that can be easily represented in a visual manner: average of the population of synapse size and intensity, inter-punctal distance and location of the most posterior synapse. In order to enhance the differences, synapses are shown in descending order according to size and intensity from left (anterior) to right (posterior). Puncta are divided in 4 groups to represent variations in size, distance and intensity.

7. Hierarchical clustering

Hierarchical clustering was performed in MATLAB ®. The data matrix used contained the mean for each z-scored (normalized as explained earlier) feature for each population. Clustering was performed on the rows only (i.e., worm populations). Pairwise distances between rows were computed with a standardized Euclidean distance according to the formula:

$$d_{ij} = \left[\sum_{k=1}^{n} (x_{ik} - x_{jk}) W^{-1} (x_{ik} - x_{jk})'\right]^{1/2}$$

Where n is the number of features (76 in this case), x_{ik} represents the mean of population i for feature k, and x_{jk} represents the mean of population j for feature k. Since the features are not independent, these were given a weight W, to avoid overestimating the importance of information more frequently computed in the phenotypic profile. The weights (W) were computed by calculating the Pearson's correlation coefficient

between features, from a matrix containing data for all the worms analyzed. Once the Pearson's correlation coefficient (PCC) was estimated between all the features, the absolute value of the PCCs for a single feature were added.

For example, the weight for feature i is estimated as:

$$W_i = \sum_{j=1}^{n} \left| PCC_{ij} \right|$$

Where PCC_{ij} is the Pearson's correlation coefficient between features i and j; and n is the total number of features. The maximum value for the weight (W) is 76, and the minimum is 0. Clustering was performed with average linkage method.

The list of genotypes in the presented hierarchical clustering figure (Main Figure 6b), in order from top to bottom, is: *a163, mtm-6(ok330), a219, a090, a208, a223, a226, a229, a224, a218, pct-1(wy575), a177, a225, a220, a207, a176, a179, a206, cdka-1(a107), Wildtype, a217, a230, a231, a222, a228, a221, a227, jnk-1(gk-7), jkk-1(km-2), a178, a178(x3), lin-44(n1792), lin-44(a175), cdk-5(ok626), cdka-1(tm648), cdk-5(a109), a115, sad-1(ky330), syd-2(wy5), cyy-1(wy302), arl-8(wy271), a085.*

8. Morphological features included in the phenotypic profile:

1) Number of puncta larger than one pixel.
2) Average size of all the puncta.
3) Average size of the puncta larger than one pixel.
4) $\frac{\text{Second central moment of the size of the puncta larger than one pixel}}{(\text{Mean of the size of puncta size larger than one pixel})^2}$
5) Number of puncta larger than 8 pixels.
6) Percentage of puncta smaller than 6 pixels.
7) $\frac{\text{Number of puncta larger than 1 pixel and smaller than 8 pixels}}{\text{Number of puncta larger than 1 pixel}}$
8) $\frac{\text{Standard deviation of the puncta size}}{\text{Mean of the puncta size}}$
9) First quartile of puncta size
10) Median of puncta size
11) Third quartile of puncta size
12) 90th percentile of puncta size
13) Maximum puncta size
14) Mean size of the smallest half of the puncta
15) $\frac{\text{Standard deviation of the size of the smallest half of the puncta}}{\text{Mean of the size of the smallest half of the puncta}}$
16) Mean size of the largest half of the puncta
17) $\frac{\text{Standard deviation of the size of the largest half of the puncta}}{\text{Mean of the size of the largest half of the puncta}}$
18) $\frac{\text{90th percentile of the puncta size}}{\text{First quartile of the puncta size}}$
19) $\frac{\text{Mean size of the largest half of the puncta}}{\text{Mean size of the smallest half of the puncta}}$

20) Mean of average puncta intensity (average puncta intensity refers to the mean pixel intensity value for each puncta)

21) $\frac{\text{Standard deviation of average puncta intensity}}{\text{Mean of average puncta intensity}}$

22) Mean of integrated puncta intensity (integrated intensity refers to the sum of all pixel intensity values for each puncta)

23) $\frac{\text{Standard deviation of integrated puncta intensity}}{\text{Mean of integrated puncta intensity}}$

24) $\frac{\text{Second central moment of integrated puncta intensity}}{(\text{Mean of integrated puncta intensity})^2}$

25) Minimum of integrated puncta intensity

26) First quartile of integrated puncta intensity

27) Median quartile of integrated puncta intensity

28) Third quartile of integrated puncta intensity

29) 90th percentile of integrated puncta intensity

30) Maximum of integrated intensity

31) Mean of integrated intensity of the third most anterior puncta

32) Mean of integrated intensity of the third central puncta

33) Mean of integrated intensity of the third most posterior puncta

34) $\frac{\text{Mean of integrated intensity of the third most posterior puncta}}{\text{Mean of integrated intensity of the third most anterior puncta}}$

35) $\frac{\text{Mean of integrated intensity of the third most posterior puncta}}{\text{Mean of integrated intensity of the third central puncta}}$

36) $\frac{\text{Mean of integrated intensity of the third central puncta}}{\text{Mean of integrated intensity of the third most anterior puncta}}$

37) Total distance of synaptic domain (computed by adding distance of individual interpunctal segments larger than 3 pixels)

38) Mean interpunctal distance (ignoring segments smaller than 3 pixels)

39) $\frac{\text{Standard deviation of interpunctal distance (ignoring segments smaller than 3 pixels)}}{\text{Mean of interpunctal distance (ignoring segments smaller than 3 pixels)}}$

40) Mean interpunctal distance of half most anterior puncta (ignoring segments smaller than 3 pixels)

41) $\frac{\text{Standard deviation of interpunctal distance of half most anterior puncta (ignoring segments smaller than 3 pixels)}}{\text{Mean of interpunctal distance of half most anterior puncta (ignoring segments smaller than 3 pixels)}}$

42) Mean interpunctal distance of half most posterior puncta (ignoring segments smaller than 3 pixels)

43) $\frac{\text{Standard deviation of interpunctal distance of half most posterior puncta (ignoring segments smaller than 3 pixels)}}{\text{Mean of interpunctal distance of half most posterior puncta (ignoring segments smaller than 3 pixels)}}$

44) 90th percentile of interpunctal distance (including all segments)

45) Density, computed by: $\frac{\text{Number of puncta larger than 1 pixel}}{\text{Total distance of synaptic domain (ignoring segments smaller than 3 pixels)}}$

46) Percentage of puncta smaller than 5 pixels

47) Percentage of puncta smaller than 10 pixels and larger or equal than 5 pixels

48) Percentage of puncta smaller than 15 pixels and larger or equal than 10 pixels

49) 95th percentile of interpunctal distance (including all segments)

50) Third quartile of interpunctal distance (including all segments)

51) Location of most posterior puncta, relative to the location of the gut end (in pixels). Negative values indicate the most posterior puncta is farther back than the gut end.

52) Mean of average interpunctal intensity *(interpunctal intensity refers to the mean pixel intensity value for each interpunctal segment)*

53) $\frac{\text{Standard deviation of interpunctal intensity}}{\text{Mean of interpunctal intensity}}$

54) 10th percentile of puncta size

55) 10th percentile of integrated intensity

56) Fraction of puncta pixels with intensity larger or equal than 500 and smaller than 1000

57) Fraction of puncta pixels with intensity larger or equal than 1000 and smaller than 1500

58) Fraction of puncta pixels with intensity larger or equal than 1500 and smaller than 2000

59) Fraction of puncta pixels with intensity larger or equal than 2000 and smaller than 2500

60) Fraction of puncta pixels with intensity larger or equal than 2500 and smaller than 3000

61) Fraction of puncta pixels with intensity larger or equal than 3000 and smaller than 3500

62) Fraction of puncta pixels with intensity larger or equal than 3500 and smaller than 4000

63) Range of puncta pixel intensity values (computed by subtracting the dimmest pixel value from the brightest pixel value)

64) $\frac{\text{Standard deviation of puncta pixel values}}{\text{Mean of puncta pixel values}}$

65) First quartile of standardized pixel values (standardized pixel values refers to the intensity pixel values where the value of the dimmest pixel has been subtracted)

66) Median of standardized pixel values

67) Third quartile of standardized pixel values

68) 90th percentile of standardized pixel values

69) Fraction of pixels with standardized intensity values smaller than 0.1(Range of pixel values)

70) Fraction of pixels with standardized intensity values smaller than 0.25(Range of pixel values) and larger than 0.1(Range of pixel values)

71) Fraction of pixels with standardized intensity values smaller than 0.5(Range of pixel values) and larger than 0.25(Range of pixel values)

72) Fraction of pixels with standardized intensity values smaller than 0.75(Range of pixel values) and larger than 0.5(Range of pixel values)

73) Fraction of pixels with standardized intensity values smaller than 0.9(Range of pixel values) and larger than 0.75(Range of pixel values)

74) Fraction of pixels with standardized intensity values smaller than 0.95(Range of pixel values) and larger than 0.9(Range of pixel values)

75) Number of puncta larger than 0.25(Range of puncta size); where range of puncta size is computed by subtracting the smallest puncta size from the largest puncta size

76) Total integrated intensity: sum of all puncta pixel intensity values

9. Genotypes used in this study:

1. Wildtype

a) Synaptic patterning known mutant collection

2. *arl-8(wy271)*
3. *cdk-5(ok626)*
4. *cdka-1(tm648)*
5. *cyy-1(wy302)*
6. *jkk-1(km-2)*
7. *jnk-1(gk-7)*
8. *lin-44(a175)*
9. *lin-44(n1792)*
10. *mtm-6(ok330)*
11. *pct-1(wy575)*
12. *sad-1(ky330)*
13. *syd-2(wy5)*

b) Previously isolated mutant collection [1]

14. *a085*
15. *a090*
16. *cdka-1(a107)*
17. *cdk-5(a109)*
18. *a115*

c) Mutants isolated in this screen

19. *a163*
20. *a176*
21. *a177*
22. *a178*
23. *a179*
24. *a206*
25. *a207*
26. *a208*
27. *a217*
28. *a218*
29. *a219*
30. *a220*
31. *a221*
32. *a222*
33. *a223*
34. *a224*
35. *a225*
36. *a226*
37. *a227*
38. *a228*
39. *a229*
40. *a230*
41. *a231*

d) *sax-2* alleles (for *a178 phenocopy experiments*)

42. *sax-2(ot10)*

43. sax-2(ky216)

10. Classification of features for pie charts (Fig. S8):

Feature	Category
1	Number
2	Size
3	Size
4	Size
5	Number
6	Size
7	Size
8	Size
9	Size
10	Size
11	Size
12	Size
13	Size
14	Size
15	Size
16	Size
17	Size
18	Size
19	Size
20	Intensity
21	Intensity
22	Cumulative Intensity
23	Cumulative Intensity
24	Cumulative Intensity
25	Cumulative Intensity
26	Cumulative Intensity
27	Cumulative Intensity
28	Cumulative Intensity
29	Cumulative Intensity
30	Cumulative Intensity
31	Cumulative Intensity
32	Cumulative Intensity
33	Cumulative Intensity
34	Cumulative Intensity
35	Cumulative Intensity
36	Cumulative Intensity
37	Distance
38	Distance
39	Distance
40	Distance
41	Distance
42	Distance
43	Distance
44	Distance
45	Distance
46	Size

47	Size
48	Size
49	Distance
50	Distance
51	Location
52	Ectopic Intensity
53	Ectopic Intensity
54	Size
55	Cumulative Intensity
56	Intensity
57	Intensity
58	Intensity
59	Intensity
60	Intensity
61	Intensity
62	Intensity
63	Intensity
64	Intensity
65	Intensity
66	Intensity
67	Intensity
68	Intensity
69	Intensity
70	Intensity
71	Intensity
72	Intensity
73	Intensity
74	Intensity
75	Number
76	Intensity

11. Imaging sets, sample sizes

Imaging experiments were performed in separate imaging rounds. Each imaging round contains a wildtype set as reference, to which normalization was performed against (see data normalization below). Imaging sets in the same round were imaged within a 2 week period.

Strain	Sample size	Imaging round
a085	144	1
a090	113	1
cdka-1(a107)	170	1
cdk-5(a109)	86	1
a115	125	1
a163	66	1

lin-44(a175)	96	1
a176	320	1
a177	134	1
a178	226	1
a179	323	1
a206	175	1
a207	139	1
a208	128	1
Wildtype	443	1
arl-8(wy271)	111	1
cdk-5(ok626)	226	1
cdka-1(tm648)	124	1
cyy-1(wy302)	132	1
jnk-1(gk7)	152	1
jkk-1(km2)	268	1
lin-44(n1792)	225	1
pct-1(wy575)	131	1
sad-1(ky330)	126	1
syd-2(wy5)	239	1
a222	64	2
a223	90	2
a224	49	2
a217	65	2
a225	74	2
a226	86	2
a227	77	2
a228	66	2
a229	55	2
a230	58	2
a218	111	2
a231	125	2
a219	90	2
a220	106	2
a221	109	2
a176-R2	91	2
a178(x3)	39	2
Wildtype	110	2
mtm-6(ok330)	73	2

Supplementary References

1. Crane, M.M. et al. Autonomous screening of *C. elegans* identifies genes implicated in synaptogenesis. *Nat Meth* **9**, 977-980 (2012).
2. Chung, K.H., Crane, M.M. & Lu, H. Automated on-chip rapid microscopy, phenotyping and sorting of *C. elegans*. *Nature Methods* **5**, 637-643 (2008).
3. Klassen, M.P. & Shen, K. Wnt Signaling Positions Neuromuscular Connectivity by Inhibiting Synapse Formation in *C. elegans*. *Cell* **130**, 704-716 (2007).
4. Cáceres, I.d.C., Valmas, N., Hilliard, M.A. & Lu, H. Laterally Orienting *C. elegans* Using Geometry at Microscale for High-Throughput Visual Screens in Neurodegeneration and Neuronal Development Studies. *PLoS ONE* **7**, e35037 (2012).
5. Chokshi, T.V., Bazopoulou, D. & Chronis, N. An automated microfluidic platform for calcium imaging of chemosensory neurons in *Caenorhabditis elegans*. *Lab on a Chip* **10**, 2758-2763 (2010).
6. Chang, C.-C. & Lin, C.-J. LIBSVM: A library for support vector machines. *ACM Trans. Intell. Syst. Technol.* **2**, 1-27 (2011).
7. Fan, R.-E., Chang, K.-W., Hsieh, C.-J., Wang, X.-R. & Lin, C.-J. LIBLINEAR: A Library for Large Linear Classification. *J. Mach. Learn. Res.* **9**, 1871-1874 (2008).
8. Vidakovic, B. Statistics for bioengineering sciences. (Springer, New York; 2011).
9. Hosmer, D.W. & Lemeshow, S. Applied Logistic Regression, Edn. 2nd. (John Wiley & Sons, Inc., New York; 2000).
10. Li, H. & Durbin, R. Fast and accurate short read alignment with Burrows–Wheeler transform. *Bioinformatics* **25**, 1754-1760 (2009).
11. Li, H. et al. The sequence alignment/map format and SAMtools. *Bioinformatics* **25**, 2078-2079 (2009).
12. https://github.com/broadinstitute/picard (2015).
13. McKenna, A. et al. The Genome Analysis Toolkit: a MapReduce framework for analyzing next-generation DNA sequencing data. *Genome research* **20**, 1297-1303 (2010).
14. Auwera, G.A. et al. From FastQ data to high‐confidence variant calls: the genome analysis toolkit best practices pipeline. *Current Protocols in Bioinformatics*, 11.10. 11-11.10. 33 (2013).
15. Cingolani, P. et al. A program for annotating and predicting the effects of single nucleotide polymorphisms, SnpEff: SNPs in the genome of Drosophila melanogaster strain w1118; iso-2; iso-3. *Fly* **6**, 80-92 (2012).
16. Davis, M.W. et al. Rapid single nucleotide polymorphism mapping in *C. elegans*. *BMC Genomics* **6**, 118 (2005).
